# Draft Genome Sequence of *Gordonia* sp. Strain UCD-TK1 (Phylum *Actinobacteria*)

**DOI:** 10.1128/genomeA.01121-16

**Published:** 2016-10-13

**Authors:** Tynisha M. Koenigsaecker, Jonathan A. Eisen, David A. Coil

**Affiliations:** a University of California Davis Genome Center, Davis, California, USA; b Department of Evolution and Ecology and Department of Medical Microbiology and Immunology, University of California Davis, Davis, California, USA

## Abstract

Here, we present the draft genome of *Gordonia* sp. strain UCD-TK1. The assembly contains 5,470,576 bp in 98 contigs. This strain was isolated from a disinfected ambulatory surgery center.

## GENOME ANNOUNCEMENT

Members of the genus *Gordonia* are Gram-positive, aerobic bacilli that are commonly isolated from soil and water (1). Previously classified in the genus *Rhodococcus*, they are frequently misidentified as such following biochemical testing (2). Some *Gordonia* species are opportunistic pathogens that have been implicated in nosocomial infections, particularly in immunocompromised patients or those with medical devices, such as catheters (1, 3).

*Gordonia* sp. strain UCD-TK1 was isolated from a patient chair in the recovery room of an ambulatory surgery center in Redding, CA, USA, as part of an ongoing undergraduate research project to provide microbial reference genomes from the built environment. The chair had been cleaned with CaviCide, an Environmental Protection Agency–approved disinfectant, prior to swabbing. A sterile cotton-tipped applicator (Puritan) was used to swab the surface of the chair and then plate the sample on lysogeny broth agar. The plate was incubated at 37°C for 5 days. Individual colonies were streaked for isolation and, once isolated, were used to make an overnight culture that was incubated at 37°C. DNA was extracted from the overnight culture following the protocol of a Promega Wizard Genomic DNA purification kit. The 16S rRNA gene was amplified using PCR with 27F and 1391R primers. DNA was then purified and used for Sanger sequencing in which DNA is replicated in the presence of dideoxynucleotides generating varying lengths of DNA sequences. Sequences are then ordered by size and base to reconstruct the original DNA sequence. The resulting consensus sequence was analyzed using BLAST (4). Top hits were aligned using the Ribosomal Database Project (5). The alignment was then used to infer a maximum-likelihood phylogenetic tree, using Fast Tree (6), which was visualized in Dendroscope (7). The organism was found in a clade containing *Gordonia terrae* and *Gordonia lacunae*, along with other unnamed species of *Gordonia*.

For whole-genome sequencing, a paired-end library was prepared using a Nextera XT library preparation kit (Illumina). We selected 600- to 900-bp fragments using a Pippin Prep (Sage Science). A portion of an Illumina MiSeq sequencing run generated 653,024 paired reads with a read length of 300 bp. After quality trimming and error correction were completed by the A5-miseq assembly pipeline (8, 9), 577,554 quality reads remained in 95 scaffolds, with 22× coverage and a GC content of 67.8%. Genome completeness was estimated using the Phylosift software (10), which searches for a list of 37 highly conserved, single-copy marker genes (11), all of which were found in this assembly in a single copy.

Annotation was performed using RAST (12). *Gordonia* sp. strain UCD-TK1 contains 5,032 coding sequences, and 64 noncoding RNAs. A partial-length 16S sequence (857 bp) was obtained from RAST and analyzed using BLAST. As expected, top hits (100% identity), included *G. terrae* and *G. lacunae*, along with unnamed *Gordonia* species. A phylogenetic tree was generated, as described above, and again was unable to resolve the taxonomy between the two strains. Two *G. terrae* whole-genome sequences have been published, but none for *G. lacunae*. Therefore, we were unable to assign a species name to this organism without further biochemical/chemotaxonomic characterization.

### Accession number(s).

This whole-genome shotgun project has been deposited at DDBJ/EMBL/GenBank under the accession number LZMP00000000. The version described in this paper is the first version, LZMP01000000.
